# Noninvasive Test for Tuberculosis Detection among Primates

**DOI:** 10.3201/eid2103.140052

**Published:** 2015-03

**Authors:** Tiffany M. Wolf, Lawrence Mugisha, Fernanda Miyagaki Shoyama, Melanie J. O’Malley, JoAnne L. Flynn, Benon Asiimwe, Dominic A. Travis, Randall S. Singer, Srinand Sreevatsan

**Affiliations:** Minnesota Zoological Gardens, Apple Valley, Minnesota, USA (T.M. Wolf);; University of Minnesota, St. Paul, Minnesota, USA (T.M. Wolf, L. Mugisha, F.M. Shoyama, D.A. Travis, R.S. Singer, S. Sreevatsan);; Conservation & Ecosystem Health Alliance, Kampala, Uganda (L. Mugisha);; Makerere University, Kampala (L. Mugisha, B. Asiimwe);; University of Pittsburgh School of Medicine, Pittsburgh, Pennsylvania, USA (M.J. O’Malley, J.L. Flynn)

**Keywords:** *Mycobacterium tuberculosis*, *africanum*, bacteria, noninvasive testing, fecal IS*6110* PCR, nonhuman primates, cynomolgus, rhesus, macaque, *Macaca fascicularis*, *Macaca mulatta* chimpanzee, *Pan troglodytes*, tuberculosis and other mycobacteria

## Abstract

Traditional testing methods have limited epidemiologic studies of tuberculosis among free-living primates. PCR amplification of insertion element IS*6110* of *Mycobacterium tuberculosis* from fecal samples was evaluated as a noninvasive screening test for tuberculosis in primates. Active tuberculosis was detected among inoculated macaques and naturally exposed chimpanzees, demonstrating the utility of this test.

The susceptibility to tuberculosis (TB) of nonhuman primates in captivity is established (*1**,**2*), although the extent of the disease among free-living primates remains unclear. Much of our understanding of primate TB is based on documentation of *Mycobacterium* *tuberculosis* transmission in captive primates (*1**,**2*), but TB caused by *M. bovis* spillover dominates among populations of free-living monkeys (*3**,**4*). Research demonstrates increases in *M. tuberculosis* complex (MTC) infections among free-ranging macaques in areas of frequent human contact and high human TB prevalence (*5*). The first evidence of TB in a free-living ape was reported in 2009 in West Africa; the infectious agent was identified upon routine necropsy of a chimpanzee as a novel MTC strain closely related to lineage 6 (i.e., *M. africanum* West-Africa type-2) (*6*).

Epidemiologic studies of TB among free-living primates have been limited by existing diagnostic technologies. Diagnosis of disease in primates traditionally relies upon procedures that identify tissue lesions or demonstrate host immune responses or upon culture of the organism (*1*), methods that are generally not feasible for free-living species because of the need for handling and anesthesia. To overcome this challenge, we evaluated a novel approach using molecular detection of MTC-specific DNA in noninvasively collected fecal samples. This approach has shown excellent sensitivity among humans with active pulmonary TB (*7**,**8*). Our objective was to evaluate the performance of PCR amplification of insertion element IS*6110* of *M. tuberculosis* in fecal samples (fecal IS*6110* PCR) for noninvasive TB detection in inoculated and naturally exposed primates.

## The Study

Fecal IS*6110* PCR was first evaluated by using samples from primates with known TB infection status. Fecal samples were collected from 41 adult (>4 years) cynomolgus macaques (*Macaca fascicularis*) included in experimental *M. tuberculosis* infection studies and 13 uninfected rhesus macaques (*M. mulatta*) included in diabetes studies. All experiments and protocols were approved by institutional animal care and use committees at the University of Pittsburgh School of Medicine or University of Minnesota.

For concurrent studies, 36 cynomolgus macaques were inoculated with a low or mid dose (≈25 or 50–100 colony-forming units, respectively) *M. tuberculosis* Erdman strain by bronchoscopic instillation, as described (*9*); 5 animals were uninfected controls. samples from 10 macaques that had active disease, 23 animals characterized as latently infected, and 3 infected animals classified as subclinically diseased or “percolators” (Technical Appendix) (*9*). Fecal samples were collected from all macaques on a single day, coinciding with varying durations of infection, ranging from 63 to 286 days (Table 1). The online Technical Appendix includes details on disease development and infection status classification.

**Table 1:** Fecal IS*6110* PCR results for detection of tuberculosis among cynomulgus and rhesus macaques, by infection status, inoculation dose, and time to sampling

Species and infection status	Inoculation dose	No. animals	Time postinoculation for sampling, mo	No. PCR positive
Cynomolgus				
Active	Mid	4	2	2
	Mid	1	5	1
	Mid	1	6	0
	Low	2	7	1
	Low	1	8	1
	Low	1	9	0
Latent	Mid	4	2	0
	Mid	1	5	0
	Mid	4	6	0
	Low	3	7	0
	Low	4	8	0
	Low	4	9	1
	Low	3	10	1
Subclinical	Low	2	7	1
	Low	1	8	0
Uninfected	N/A	5	NA	0
Rhesus				
Uninfected	N/A	13	NA	0

Fecal IS*6110* PCR was also evaluated in primates under conditions of natural exposure and infection. Fecal samples were collected from 36 juvenile and adult (7–27 y, mean 15 y) chimpanzees (*Pan troglodytes*) managed in 2 sanctuaries and 1 zoo in East Africa. Housing and management are described in the online Technical Appendix. All animals were considered to be clinically healthy during sampling. Fecal PCR results of sanctuary chimpanzees were compared with their most recent tuberculin skin test (TST) responses (*10*). TSTs were performed opportunistically on 27 chimpanzees during routine exams on the same day as fecal collection. For the remaining 9 animals, TST results were available from 9 months before fecal collection for 3 chimpanzees and from 2 years before for 6 chimpanzees. In addition to TST, results from the PrimaTB Stat-PAK (Chembio Diagnostic Systems, Inc., Medford, New York, USA), a field-based serologic assay, were also available for 6 animals.

We extracted DNA from fecal samples using the QIAamp DNA Stool Mini Kit (Qiagen, Inc., Valencia, CA, USA). Feces-free negative controls were included in all extraction procedures. Conventional and real-time PCR were used to amplify a portion of the IS*6110* insertion sequence. Primers, master mixes, and thermocycling conditions are included in Table 2. For conventional PCR, amplicons of target size were confirmed as IS*6110* by Sanger sequencing (University of Minnesota Genomics Center, St. Paul, Minnesota, USA). Nuclease-free water (QIAGEN) negative controls were included in all amplification reactions. The Technical Appendix contains additional methodological details.

**Table 2:** Fecal IS*6110* conventional and real-time PCR master mixes and reaction conditions for investigation of noninvasive tuberculosis detection in primates

PCR type	Primers, 5′ → 3′	Master mix	Reaction conditions
Conventional	Forward: TTCAGGTCGAGTACGCCTTC Reverse: CGAACTCAAGGAGCACATCA	12.5 μL HotStarTaq Master Mix:* 8 μL RNase-free water,* 0.4 μM of each primer, 1.25 μL DMSO, 0.25 μL 1% BSA, 1 μL DNA template. Total volume 25 μL	95°C for 15 min/DNA polymerase activation; 40 cycles: 94°C for 30 s/denaturation, 56°C for 30 s/annealing, 72°C for 1 min/extension. Termination at 72°C for 10 min
Real-time	Forward: AGAAGGCGTACTCGACCTGA Reverse: CCGGATCGATGTGTACTGAG	LightCycler 480 Probes Master:† 0.2 mM of each primer, 0.2 mM of the FAM-labeled IS*6110* probe,† 5 μL DNA template. Total volume 25 μL	95°C for 5 min; 45 cycles: 95°C for 10 s/denaturation; 50°C for 30 s/annealing; 72°C for 1 s/extension. Termination at 65°C–95°C at 2.2°C/s/melting curve analysis

*QIAGEN, Inc., Valencia, CA, USA. †Roche, Indianapolis, IN, USA

## Conclusions

Fecal IS*6110* PCR was effective in identifying 5 of 10 inoculated macaques with active disease and 8 of 36 total infected macaques. No uninoculated macaques were positive by results of IS*6110* PCR. Conventional PCR identified 3 actively infected macaques and real-time PCR identified 2 additional active infections. Two latently infected macaques and 1 with subclinical infection were also positive by using IS*6110* PCR. Overall sensitivity for this testing method was 22% (95% Wilson CI 12%–38%) and specificity was 100% (95% Wilson CI 82%–100%). Sensitivity of detection of active infections was estimated at 50% (95% Wilson CI 24%–76%). The latter sensitivity estimate is equivalent to that of gastric aspirate of children with radiographic evidence of pulmonary TB (*11*). 

The observed sensitivity of fecal IS*6110* PCR is limited by several factors. Unlike immunologic tests, the success of this approach relies on bacterial shedding in sputum, subsequent swallowing, and excretion in feces; hence, active infection. Thus, most latent infections may go undetected, as observed in this study. Aside from outbreaks, identifying large numbers of actively infected primates for test validation is challenging. We sampled animals in experimental infection studies, but even so, active infections were few. Also, low numbers of organisms are likely shed intermittently in feces; thus, serial testing of multiple fecal samples may improve diagnostic sensitivity. PCR may also be paired with mycobacterial culture of feces for further molecular characterization of infection (*8*). Overall, this study demonstrates that fecal detection of mycobacterial DNA is best suited for identifying actively infected primates, which are crucial in TB transmission.

TST conversion was not observed in any chimpanzees; however, IS*6110* DNA was detected in 3 chimpanzee fecal samples. TST was conducted the same day as fecal sampling for 1 of these animals, 9 months before for 1 animal, and 2 years before for 1 animal. TST is a common TB screening method used in primate sanctuaries but it is limited by sensitivity and specificity (*1*). Although this limitation can be overcome with Bayesian methods to estimate sensitivity and specificity for test validation purposes, the challenge remains in effectively identifying populations of captive primates with TB. Unfortunately, confirmation of infection status by additional diagnostic testing modalities of the 3 fecal PCR–positive chimpanzees has been limited. 

Test results for 1 fecal PCR–positive chimpanzee demonstrated an immunological response to *M. tuberculosis* antigen by using the PrimaTB Stat-Pak, but culture of a bronchoalveolar lavage (BAL) sample was unsuccessful. Another chimpanzee, positive by fecal PCR, retested positive the next year by fecal IS*6110* PCR. The body size of this 14-year-old male that was historically TST negative was stunted (e.g., reduced growth) compared with other male chimpanzees of similar age. 

These circumstances demonstrate the complexity of TB diagnosis and the challenges surrounding successful validation of TB tests in the natural setting. To reach a more complete understanding of diagnostic performance of fecal IS*6110* PCR in a natural setting where disease prevalence is low, large-scale and long-term testing across many captive primate populations is still needed.

Fecal IS*6110* PCR is a novel approach to the noninvasive detection of TB infection in primates, offering a new opportunity to screen for TB in free-living primates. IS*6110* detection is advantageous for its MTC specificity, which is optimal given the known susceptibility of primates to *M. bovis*, *M. tuberculosis*, and the recently discovered strain known as chimpanzee bacillus. This approach offers new direction for the epidemiologic investigation of tuberculosis in free-living primate populations.

Technical Appendix Additional methods on study design, infection status confirmation in inoculated macaques, and laboratory techniques.
