# Supplementary material for: Noninvasive Test for Tuberculosis Detection among Primates
Source: Emerg Infect Dis. 2015 Mar;21(3):468–70. doi: 10.3201/eid2103.140052 (PMC4344255; doi:10.3201/eid2103.140052)
Supplement: Technical Appendix — Additional methods on study design, infection status confirmation in inoculated macaques, and laboratory techniques. [file 14-0052-Techapp-s1.pdf]

DOI: http://dx.doi.org/10.3201/eid2103.140052

Noninvasive Test for Tuberculosis Detection in Primates

Technical Appendix

Additional Methods

Fecal samples for IS*6110* PCR were collected from 41 adult (>4 years) cynomolgus macaques (*Macaca fascicularis*) included in experimental *M. tuberculosis* infection studies and 13 uninfected rhesus macaques (*Macaca mulatta*) included in diabetes studies. Uninfected animals were housed under BSL-2 conditions; infected animals were housed in BSL-3 level facilities.

Thirty-six cynomolgus macaques were infected with a low or mid dose (≈25 or 50–100 colony-forming units, respectively) *M. tuberculosis* Erdman strain by bronchoscopic instillation, as described (*1*). Infection was confirmed by tuberculin skin test (TST) conversion, lymphocyte proliferation assay, and gamma interferon enzyme-linked immunosorbent spot of peripheral blood mononuclear cells (PBMC) (*1*). In the cynomolgus macaque low-dose model, half of the animals develop active tuberculosis, typically 4–12 months post-infection, with substantial variability in timing and presentation of clinical signs. (*1*) The other half develop clinically latent infection and have no signs of disease. Included in this study were ten macaques that had developed active disease, as determined by clinical signs and disease on thoracic radiography in association with *M. tuberculosis* growth from gastric aspirate (GA) or bronchoalveolar lavage (BAL). *M. tuberculosis* culture methods have been described previously. (*1*) Briefly, GA sample culture was performed by the University of Pittsburgh Medical College clinical microbiology laboratory. The samples were subcultured onto LJ slants for *M. tuberculosis*. BAL samples were cultured on 7H11 plates. Twenty-three animals were characterized as latently infected based on absence of both clinical signs and *M. tuberculosis* growth with a positive TST reaction and peripheral responses to *M. tuberculosis* antigen (*1*). Three infected animals were classified as subclinically diseased based on the absence of clinical disease but intermittent *M. tuberculosis* growth on GA or BAL (*1*).

Fecal samples were collected from 36 juvenile and adult (7–27yrs, mean=15yrs) chimpanzees (*Pan troglodytes*) managed in two chimpanzee sanctuaries and one zoo in East Africa. Many chimpanzees were raised from infant stage by the sanctuaries as a result of confiscation. All are managed in social groups in indoor/outdoor housing. Human contact includes direct, intimate contact up until age 5yrs, followed by protected, close contact at older ages with regular visitation by tourists and researchers. Contact with free-ranging primate species is variable. TST is a routine tuberculosis screening test employed by the chimpanzee sanctuaries during routine examinations, offering a platform for comparison of the fecal IS*6110* PCR. In general, any TST responders are followed up with additional tuberculosis diagnostic testing (e.g. culture of BAL or GA samples).

Fecal samples were stored frozen at -80°C for 1 week to 30 months until processing. DNA was extracted using the QIAamp DNA Stool Mini Kit (QIAGEN, Inc.) according to manufacturer's instructions, with a few exceptions. First, feces (180-220mg) was added to a 2.0ml microcentrifuge tube containing 0.1mm Zirconia/Silica beads (BioSpec Products, Inc.) in addition to 1.4ml ASL Buffer, which was vortexed for 3 minutes. Additionally, water bath temperatures were increased from 70°C to 95°C. These alterations were to optimize mycobacterial cell wall lysis. Following completion of the extraction protocol, samples were stored frozen at -20°C until PCR testing.

A portion of the IS*6110* insertion sequence was amplified by conventional and real-time PCR. Primers, master mixes, and thermocycling conditions are included in Table 2. For conventional PCR, amplicons were visualized under UV illumination after electrophoresing through a 1% agarose gel containing ethidium bromide; products of target size were confirmed as IS*6110* by Sanger sequencing (University of Minnesota Genomics Center, St. Paul, Minnesota, USA).

Reference

1. Lin PL, Rodgers M, Smith L, Bigbee M, Myers A, Bigbee C, et al. Quantitative comparison of active and latent tuberculosis in the cynomolgus macaque model. [Internet]. Infect Immun. 2009;77:4631–42 PubMed http://dx.doi.org/10.1128/IAI.00592-09
